# Genome-wide association study identifies candidate genes associated with leaf morphological traits in grape (*Vitis vinifera* L.)

**DOI:** 10.3389/fpls.2026.1930463

**Published:** 2026-09-30

**Authors:** Zhongyi Yang, Tao Jiang, Congling Fang, Liru Wang, Yuxuan Zhou, Yangshengkai Xu, Jiali Miao, Yueyan Wu

**Affiliations:** 1 College of Biology and Environment, Zhejiang Wanli University, Ningbo, China; 2 Cixi Forestry and Specialty Technology Promotion Center, Ningbo, China

**Keywords:** candidate genes, genome-wide association study, leaf morphological traits, leaf serration, *Vitis vinifera* L.

## Abstract

Grape leaf serration morphology is a key physiological trait that influences photosynthetic efficiency, leaf boundary layer dynamics, and stress adaptability, thereby affecting yield and quality. In this study, we phenotyped 10 mature leaf morphological traits in 153 grape accessions over two consecutive years. Among these traits, five showed high interannual stability (|r| ≥ 0.8). GWAS of these five stable traits using three models (GLM, MLM, and FarmCPU) identified seven high-confidence SNP loci reproducibly associated with mature leaf serration morphology. Ten candidate genes were screened within ±15 kb of these SNPs, including genes related to ubiquitin-mediated protein degradation (Cullin-4), ubiquitin ligase activity (SP-RING-type protein), and two-component signaling (histidine kinase). Based on their predicted roles in leaf development and cell differentiation, we selected four genes for qRT-PCR validation. We observed differential expression patterns associated with serration morphological variation: Cullin-4 and SP-RING-type protein were upregulated in straight-sided serrations, whereas histidine kinase was upregulated in convex-sided serrations. These expression patterns provide preliminary evidence that ubiquitin-proteasome pathway activity and phosphorylation signaling may be associated with serration variation, although direct functional validation is required. This study characterizes the genetic architecture of grape leaf serration morphology and suggests possible differential regulatory mechanisms involving protein degradation and signaling pathways, providing a foundation for future functional studies.

## 1 Introduction

Grapevine (*Vitis vinifera* L.) is a globally important fruit crop with high economic value. Improving grape quality and yield is a long-standing central goal of agricultural research, and dissecting the genetic basis of key agronomic traits underpins efficient breeding programs. As the primary photosynthetic organs, leaves are critical targets for grape genetic improvement, as they shape canopy architecture, drive energy assimilation, and determine overall plant adaptability (Zhu et al., 2010). Grape leaf morphology covers multiple features, including leaf size, serration characteristics, and cross-sectional shape, which collectively modulate physiological performance and stress responses (Demmings et al., 2019).

With advances in genomics and phenomics, the genetic basis of leaf traits has been increasingly characterized (Chitwood et al., 2016). Leaf size is a polygenic trait with strong genotype-by-environment interactions, driven by divergent environmental response strategies across genotypes (Gonzalez et al., 2012; Rice et al., 2020). In grapevine, auxin signal transduction genes have been confirmed to regulate final leaf size (Gao et al., 2021). Leaf serration (shape, depth, and density) is a defining morphological feature of grape leaves (Demmings et al., 2019) and has been suggested to influence transpiration efficiency, heat dissipation, and herbivory resistance in some plant species (Carmona et al., 2011; Nicotra et al., 2011). However, grapevine leaves differ markedly in texture and serration morphology from the deeply lobed, sharply serrated leaves of model plants; therefore, whether these functional associations can be directly extrapolated to grapevine remains to be established. Serration development relies on precise spatiotemporal regulation of marginal meristem activity, involving NAC transcription factors and auxin signaling (Kawamura et al., 2010). Deepened serration has been associated with improved water retention under drought via altered stomatal distribution in leaf margins (Hepworth et al., 2015), and CUC2-like transcription factors modulate serration depth under water deficit by integrating auxin signaling and cell wall remodeling (Bilsborough et al., 2011).

Leaf cross-sectional morphology, such as flat, V-shaped, or curled, modulates leaf orientation and thereby affects canopy light distribution, ventilation, photosynthetic efficiency, and microclimate. Moderate upward leaf rolling is an adaptive response to high temperature, strong light, and water deficit, documented in grapevine and other plant species (Koini et al., 2009; Zou et al., 2011; Tzortzakis et al., 2020). This trait is mainly regulated by asymmetric cell division and expansion between the adaxial and abaxial leaf surfaces, with auxin-related pathways involved in leaf movement and rolling (Koini et al., 2009).

GWAS has been widely applied to dissect complex quantitative traits (Xiao et al., 2022; Zhang et al., 2025) and to fruit quality traits in grapevine and other perennial fruit crops (Migicovsky et al., 2021; Zhong et al., 2021; Lin et al., 2024; Minamikawa et al., 2024; Shi et al., 2024; Wang et al., 2025); however, investigations of grape leaf traits remain limited. Most leaf morphological traits are quantitative and polygenic, and challenges in accurate phenotyping have likely slowed progress in this field. Traditional breeding has long prioritized fruit traits as direct selection targets, resulting in insufficient genetic dissection of leaf morphology—an “indirect trait” that drives yield and quality via source–sink interactions. Given that leaf morphology directly determines light capture efficiency, canopy microclimate, and stress tolerance (Zhang et al., 2024), systematic genetic dissection of grape leaf traits will fill a key gap in grapevine genomic research, provide candidate targets for breeding stress-resistant varieties with high light-use efficiency, and expand our understanding of source–sink coordination networks in perennial fruit crops. Regardless of the physiological consequences, dissecting the genetic architecture of grapevine leaf serration is a necessary first step toward understanding leaf morphological diversity and its potential agronomic relevance.

To address this gap, we initially evaluated ten key leaf morphological traits in a diverse panel of 153 grapevine accessions using whole-genome resequencing data. We first assessed their interannual stability and selected five traits with high repeatability for subsequent genome-wide association analysis. We then focused candidate gene mining on traits showing robust and reproducible signals across multiple models and years. We identified stable genetic loci and prioritized candidate genes associated with leaf serration, dissection, and cross-sectional morphology. These findings provide genetic insights into grape leaf architecture and may inform future genetic improvement strategies, although their practical application will require further evaluation.

## 2 Materials and methods

### 2.1 Plant materials

Leaf samples were collected from 153 grapevine accessions at the Grapevine Germplasm Repository of Zhejiang Wanli University (N 30.26434°, E 121.40831°) in July 2023 and July 2024. Sampling was performed between 9:00 and 12:00. Healthy, pest- and disease-free shoots of uniform maturity were selected, and mature leaves from the 7th to 9th nodes were collected. This sampling scheme followed the national agricultural industry standard of the People’s Republic of China, “NY/T 2563-2014, Guidelines for the assessment of distinctness, uniformity and stability—Grapevine (*Vitis vinifera* L.)” (NY/T 2563-2014, Guidelines for the conduct of tests for distinctness, uniformity and stability—Grapevine (Vitis L.), 2014), which specifies fully expanded leaves from the 7th to 9th nodes as representative of an accession’s typical leaf morphology. Three leaves were sampled per accession, each from a different plant of the same accession, to provide biological replicates. The same vines were used for phenotypic sampling in both growing seasons (2023 and 2024). Complete phenotypic data for the 10 traits across both growing seasons are presented (**Supplementary Table S1**). The region has a subtropical monsoon climate, with an annual mean temperature of 18.5 °C, annual precipitation of 1119.3 mm, and annual sunshine duration of 1879.2 h. The soil is brown, with a pH of 8.58 (slightly alkaline), an organic matter content of 5.44 g/kg, a water content of 1.6%, and a particle density of 2.56 g/cm³. To further characterize the environmental conditions during the two growing seasons, monthly mean temperature and precipitation records for 2023 and 2024 were obtained from the local meteorological station. Accumulated growing degree days (GDD, base 10 °C) from April to July were approximately 1651 °C·d in 2023 and 1627 °C·d in 2024, while total precipitation over the same period was 749 mm and 763 mm, respectively. These values indicate that the two growing seasons had broadly similar thermal and moisture conditions, supporting the comparability of the phenotypic data between years. To characterize the baseline tissue-specific expression profiles of the candidate genes, we used ‘Yinhong’, a stable, laboratory-developed accession maintained under uniform cultivation practices; this minimized environmental and genetic background noise and allowed precise identification of organ-specific expression patterns. To validate the genetic associations and confirm expression differences across phenotypes, nine accessions representing distinct serration morphologies were then selected for quantitative reverse-transcription PCR (qRT-PCR) analysis: three with mixed-type serrations (‘Tiangongmoyu’, ‘Yongfeihong’, and ‘Yongzaohong’), three with straight-sided serrations (‘Zaocuixiangti’, ‘Conglinmeigui’, and ‘Baiaolin’), and three with convex-sided serrations (‘Hongbaladuo’, ‘Heibaladuo’, and ‘Puzhimeng’). The serration type of each accession was determined according to the description and grading criteria of the national agricultural industry standard NY/T 2563-2014 (NY/T 2563-2014, Guidelines for the conduct of tests for distinctness, uniformity and stability—Grapevine (Vitis L.), 2014). These accessions were specifically chosen because they capture the extreme phenotypic variation in leaf serration present in our germplasm collection and represent the major genetic diversity of grapevine leaf serration traits in East Asia; we acknowledge, however, that functional validation in other grape cultivars will be necessary to extend these findings to global viticulture. All accessions were managed under conventional cultivation practices. All samples were immediately frozen in liquid nitrogen after collection and stored at −80 °C for subsequent RNA extraction.

### 2.2 Phenotypic variation of ten grapevine leaf morphological traits

The ten leaf morphological traits measured in this study were mature leaf size (YDX), mature leaf shape (YXZ), number of mature leaf lobes (YLP), serration length (JCC), serration length-to-width ratio (JCB), serration morphology (JCXZ), upper sinus depth (SLPSD), upper sinus overlapping type (SLPKD), lower sinus overlapping type (XLPKD), and leaf cross-sectional morphology (HJM). Following the description and grading criteria of the national agricultural industry standard NY/T 2563-2014 (NY/T 2563-2014, Guidelines for the conduct of tests for distinctness, uniformity and stability—Grapevine (Vitis L.), 2014), each trait was divided into 3 to 5 grades and digitally coded (**Table 1**). For each accession, three leaves, each collected from a distinct plant, served as biological replicates.

**Table 1:** Assessment criteria and scoring system for ten grapevine leaf morphological traits.

Trait	Description
Mature leaf size (YDX)	Very small = 1, Small = 3, Medium = 5, Large = 7, Very large = 9
Mature leaf shape (YXZ)	Cordate = 1, Subtriangular = 2, Subpentagonal = 3, Subcircular = 4, Reniform = 5
Number of mature leaf lobes (YLP)	Absent = 1, Trilobed = 2, Pentalobed = 3, Heptalobed = 4, More than seven lobes = 5
Mature leaf serration length (JCC)	Short = 1, Medium = 3, Long = 5
Mature leaf serration length-to-width ratio (JCB)	Very small = 1, Small = 3, Medium = 5, Large = 7, Very large = 9
Mature leaf serration morphology (JCXZ)	Convex on both sides = 1, Straight on both sides = 2, Concave on both sides = 3, Convex on one side and concave on the other = 4, Mixed straight and convex = 5
Mature leaf upper sinus depth (SLPSD)	Very shallow = 1, Shallow = 3, Medium = 5, Deep = 7, Very deep = 9
Mature leaf upper sinus overlapping type (SLPKD)	Open = 1, Closed = 2, Overlapping = 3
Mature leaf lower sinus overlapping type (XLPKD)	Open = 1, Closed = 2, Overlapping = 3
Mature leaf cross-sectional morphology (HJM)	Flat = 1, V-shaped = 2, Involute = 3, Revolute = 4, Wavy = 5

### 2.3 GWAS

Whole-genome resequencing of the 153 grapevine accessions was performed specifically for this study, with an average sequencing depth of 11.15× (range, 9.60–11.69×). Variant calling and quality control followed a previously described pipeline (Hu et al., 2025). Reads were aligned to the grapevine reference genome (*Vitis vinifera* 12X.v2) using BWA-MEM, and variants were called with GATK (v4.2.6.1) HaplotypeCaller. Low-quality SNPs were removed based on the following criteria: QD < 2.0, FS > 60.0, MQ < 40.0, MQRankSum < −12.5, ReadPosRankSum < −8.0, and deletion rate > 0.05. The raw whole-genome resequencing data and GWAS VCF files have been deposited in the Genome Sequence Archive (GSA) at the National Genomics Data Center (NGDC) under accession number PRJCA026367 (https://ngdc.cncb.ac.cn/). After initial variant calling, 58,926,380 single-nucleotide polymorphism (SNP) markers were identified. After removing loci with minor allele frequency (MAF) < 0.05 or marked as filtered, 5,848,986 high-quality SNPs were retained. After further excluding SNPs with unknown chromosomal positions, 5,620,388 SNPs were used for downstream genome-wide association analysis.

GWAS was performed using the GAPIT package in R to evaluate associations between SNPs and the five high-stability leaf morphological traits. To control for false positives and improve mapping resolution, three models were adopted: the generalized linear model (GLM), the mixed linear model (MLM), and the fixed and random model circulating probability unification (FarmCPU). MLM accounted for population structure and kinship, while FarmCPU was used to improve statistical power and reduce false positives.

Results were visualized using Manhattan plots and Q-Q plots, with the X-axis representing chromosomal position and the Y-axis showing the −log_10_(P) value. To account for multiple testing, we first calculated the Bonferroni-corrected significance thresholds as −log_10_(0.05/N) and −log_10_(0.01/N), where N represents the total number of SNPs used in the association analysis (5,620,388). These calculations gave values of 7.43 and 8.13, respectively. Considering that Bonferroni correction is highly conservative, and because we used three complementary models with two-year replication and required SNPs to be consistently detected across models and years, we adopted an empirical significance threshold of −log_10_(P) = 5, which is commonly used in grapevine GWAS. SNPs with −log_10_(P) ≥ 5 were therefore considered significantly associated with leaf morphological traits.

### 2.4 Gene screening and annotation

To reduce false positives, we used three models (GLM, MLM, and FarmCPU) to analyze the 2023 and 2024 GWAS results separately and identified stable SNP loci significant in both years. Candidate genes were screened within ±15 kb genomic intervals flanking these stable SNP loci. This window was selected because a previous study using the same 153 grapevine accessions reported that the average linkage disequilibrium (LD) decay distance was approximately 15 kb (Hu et al., 2025), indicating that ±15 kb covers most linked variants and potential candidate genes. All annotated genes within these intervals were included in the candidate gene set to capture potential cis-regulatory regions and coding genes.

For functional annotation, candidate genes were characterized using multiple databases and tools. First, basic gene information was obtained from the annotation file of the grapevine reference genome (PN40024 12X.v2). Second, protein functional annotation and subcellular localization prediction were performed using the UniProt database (https://www.uniprot.org/id-mapping, accessed on 15 June 2025). In parallel, metabolic pathway enrichment analysis was conducted using the KEGG PATHWAY database (https://www.kegg.jp/kegg/pathway.html, accessed on 15 June 2025), and TBtools-II (v2.326) was used to identify KEGG pathways significantly enriched among the candidate genes, revealing their potential roles in leaf morphogenesis and serration differentiation.

### 2.5 RNA extraction, reverse transcription, and qRT-PCR

Total RNA was extracted from leaf samples using the Polysaccharide and Polyphenol Plant RNA Rapid Extraction Kit (HuiLing, Zhenjiang, Jiangsu, China). First-strand cDNA was synthesized using the 2 × NovoScript^®^ Plus First-Strand cDNA Synthesis SuperMix (Novoprotein, Suzhou, Jiangsu, China). Gene-specific primers for qRT-PCR validation were designed using Primer Premier 5. Primer specificity was confirmed by melting-curve analysis, and amplification efficiency was determined using standard curves generated from 10-fold serial dilutions of pooled cDNA; all primer pairs showed efficiencies ranging from 99.8% to 100.2% (R^2^ ≥ 0.9993), with detailed primer sequences, amplification efficiencies, and R^2^ values provided (**Supplementary Table S2**). The *V. vinifera ACTIN* (*Vvi-ACT*) gene was used as the internal reference (Yang et al., 2026). qRT-PCR was performed using 2 × NovoStart^®^ SYBR qPCR SuperMix Plus (Novoprotein, Suzhou, China), and relative expression levels were calculated using the 2^−ΔΔCt^ method. Each qRT-PCR analysis included three independent biological replicates and three technical replicates.

### 2.6 Statistical analysis

Results are presented as mean ± standard error (SE). Statistical differences and correlation analyses were performed using one-way analysis of variance (ANOVA) followed by Tukey’s honestly significant difference (HSD) test in IBM SPSS Statistics 26.0 (IBM Corp., Armonk, NY, USA).

## 3 Results

### 3.1 Five leaf traits show high interannual stability

We first characterized the phenotypic variation and interannual stability of the ten leaf morphological traits. The median, standard deviation (SD), and coefficient of variation (CV) for each trait differed only minimally between 2023 and 2024, indicating good consistency of the phenotypic data between years (**Supplementary Table S3**). However, traits differed markedly in their patterns of variation. CV values ranged from 17.4% to 125.7%. The CV of mature leaf size (YDX) exceeded 120%, suggesting high genetic diversity in this population, whereas the CV of the number of mature leaf lobes (YLP) was below 20%, indicating a relatively narrow phenotypic distribution. Three traits—mature leaf size (YDX), serration morphology (JCXZ), and lower sinus overlapping type (XLPKD)—showed skewness > 1.0 and kurtosis > 1.0, indicating right-skewed, leptokurtic distributions. By contrast, serration length (JCC), upper sinus depth (SLPSD), and upper sinus overlapping type (SLPKD) showed negative kurtosis, consistent with relatively flat (platykurtic) phenotypic distributions. Interannual correlation analysis revealed marked differences in repeatability among traits (**Figure 1**). Five traits—YDX, JCB, JCXZ, SLPKD, and HJM—showed high interannual repeatability (|r| ≥ 0.8), indicating that they were less affected by year-to-year environmental fluctuations and are likely under stronger genetic control. The remaining traits showed lower interannual repeatability, suggesting that they may be co-regulated by environmental factors and minor-effect polygenes.

**Figure 1:**
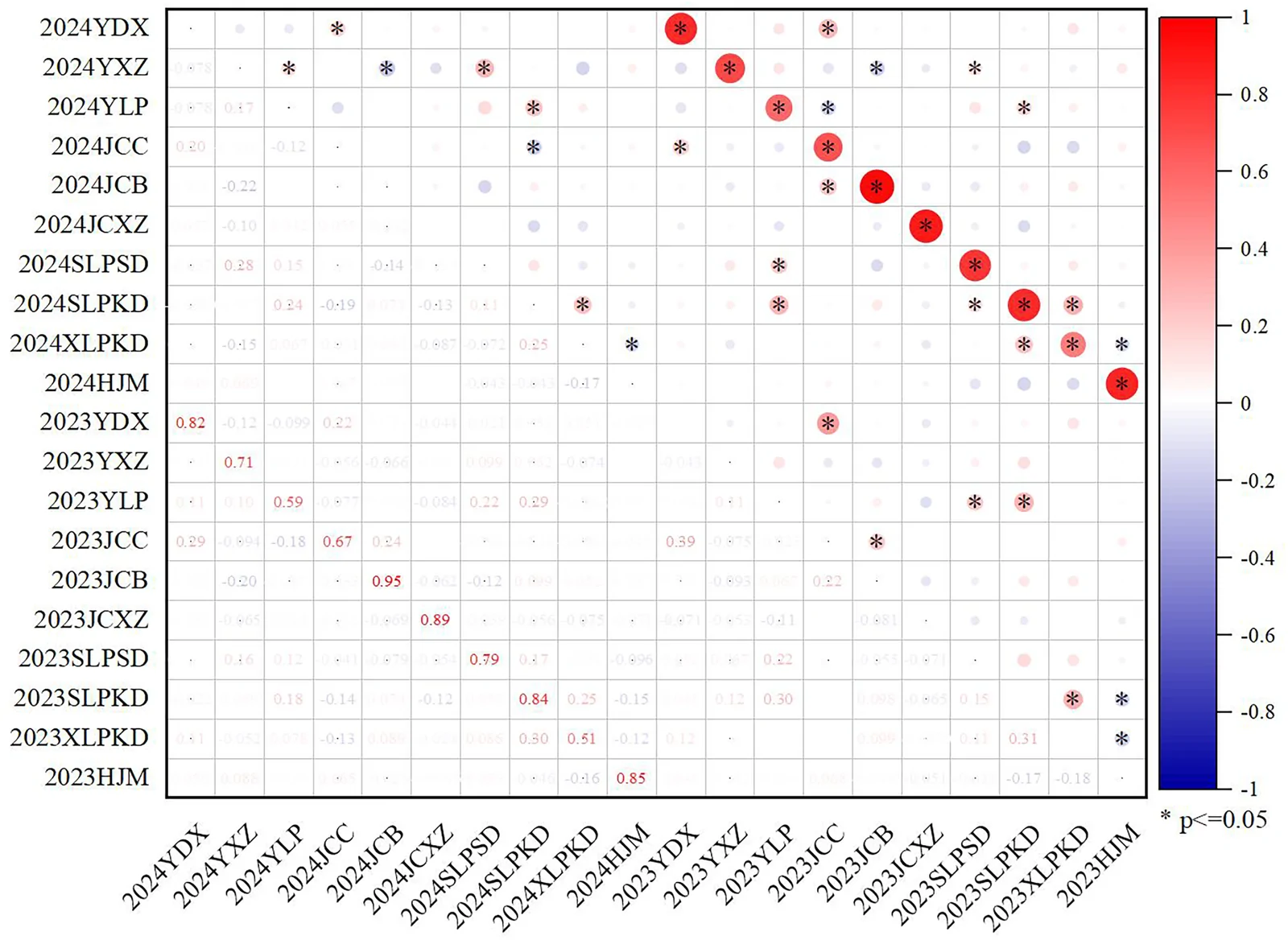
Correlation heatmap of grapevine leaf traits in 2023 and 2024. Colors represent correlation coefficients, ranging from −1 (perfect negative correlation) to 1 (perfect positive correlation), with 0 indicating no correlation. Asterisks denote statistically significant correlations (P ≤ 0.05).

Five traits showed high interannual stability and were selected for further analysis (**Figure 2**). YDX showed an upward trend in the proportion of larger-leaf categories (**Figure 2A**). For JCB, the proportion of the “very large” category varied more between years (**Figure 2B**). JCXZ remained highly stable across years and was least affected by environmental fluctuations (**Figure 2C**). For SLPKD, the proportion of overlapping types increased (**Figure 2D**). For HJM, the proportions of V-shaped and involute types increased, leading to greater morphological diversity (**Figure 2E**). We then used these five traits for genome-wide association analysis.

**Figure 2:**
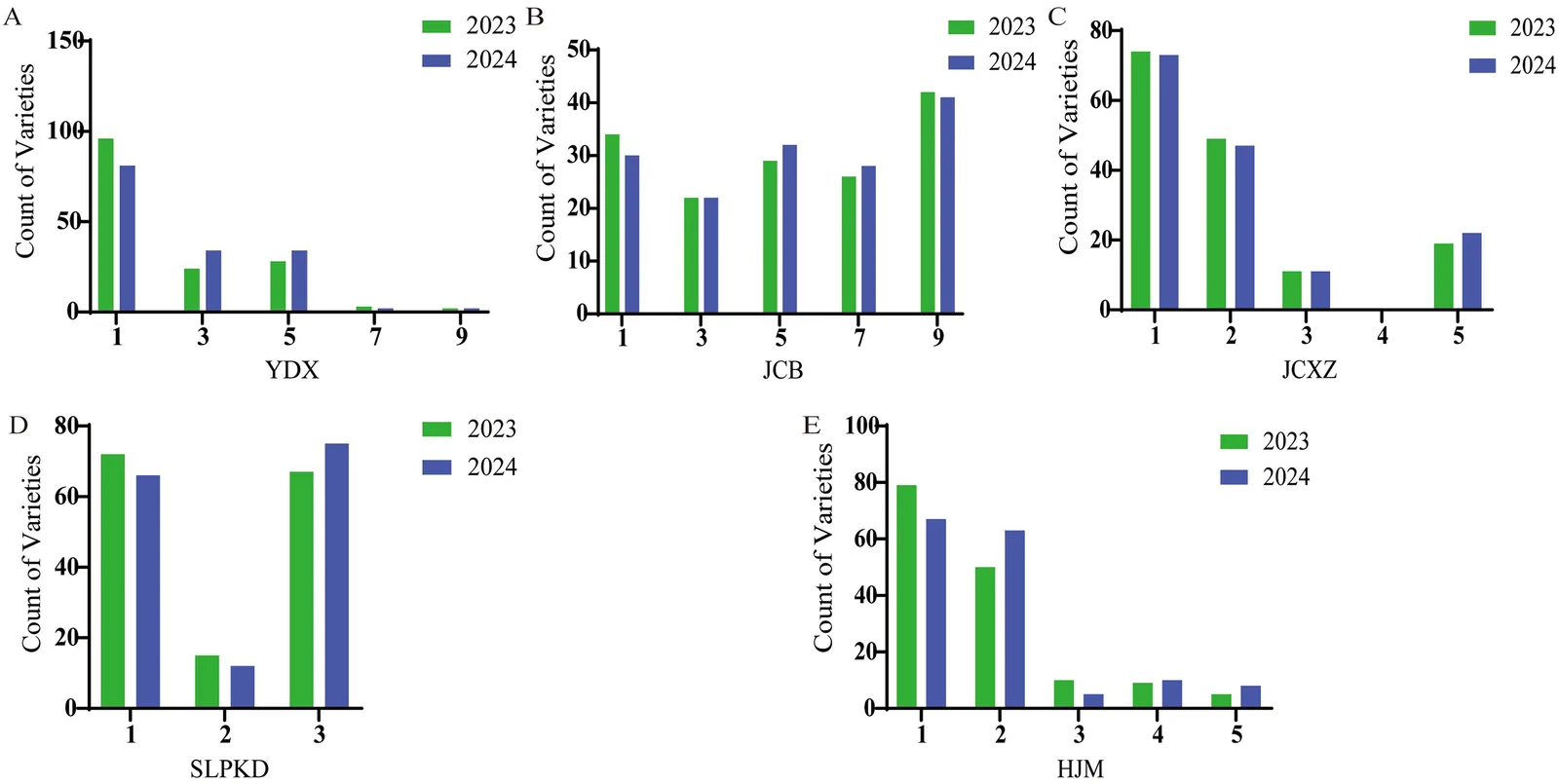
Phenotypic frequency distributions of five grapevine leaf morphological traits across two growing seasons. Histograms show the distributions of **(A)** mature leaf size (YDX), **(B)** serration length-to-width ratio (JCB), **(C)** serration morphology (JCXZ), **(D)** upper sinus overlapping type (SLPKD), and **(E)** leaf cross-sectional morphology (HJM) in 2023 (green bars) and 2024 (blue bars). The x-axis represents phenotypic categories defined by the NY/T 2563–2014 standard, and the y-axis indicates the number of accessions. A total of 153 accessions were evaluated for each trait in each year according to the grading criteria (**Table 1**).

### 3.2 Multi-model GWAS detects stable SNP loci for leaf serration traits

Based on the interannual stability analysis, five traits with |r| ≥ 0.8 were selected for GWAS because they were expected to be under stronger genetic control and therefore more suitable for association mapping. These five traits were analyzed in parallel using three complementary models (GLM, MLM, and FarmCPU), with GWAS performed separately for the 2023 and 2024 phenotypic datasets. Manhattan plots for the three models revealed marked differences in the genomic distribution of significant SNPs associated with leaf morphological traits (**Figure 3**; **Supplementary Figures S1**, **S2**). GLM results for all five traits are presented in **Figure 3**, whereas MLM and FarmCPU identified significant associations with leaf serration traits (**Supplementary Figures S1**, **S2**). The observed and expected P values were in good agreement for all traits and years, indicating that the GWAS models were appropriate (**Supplementary Figures S3**, **S4**). For JCXZ, the observed and expected P values showed no systematic deviation across all three models and both years (**Supplementary Figure S3**). For the remaining four traits (YDX, JCB, SLPKD, and HJM), the observed and expected P values also showed good agreement under FarmCPU across both years (**Supplementary Figure S4**). Across the two-year datasets, we identified 468 SNPs significantly associated with the target leaf traits, of which 306 were detected in 2023 and 162 in 2024. The number of significant SNPs detected for each trait varied across traits and models (**Supplementary Table S4**). Detailed information on all significant SNPs, including chromosomal location and physical position, is provided (**Supplementary Table S5**). Comparing detection performance across the three models, FarmCPU identified more significantly associated loci than GLM and MLM for most traits, with particularly strong detection power for serration morphology (JCXZ) and serration length-to-width ratio (JCB). In contrast, MLM detected the fewest significant loci, indicating that it imposed more stringent correction for population structure and kinship, thus effectively reducing false-positive associations.

**Figure 3:**
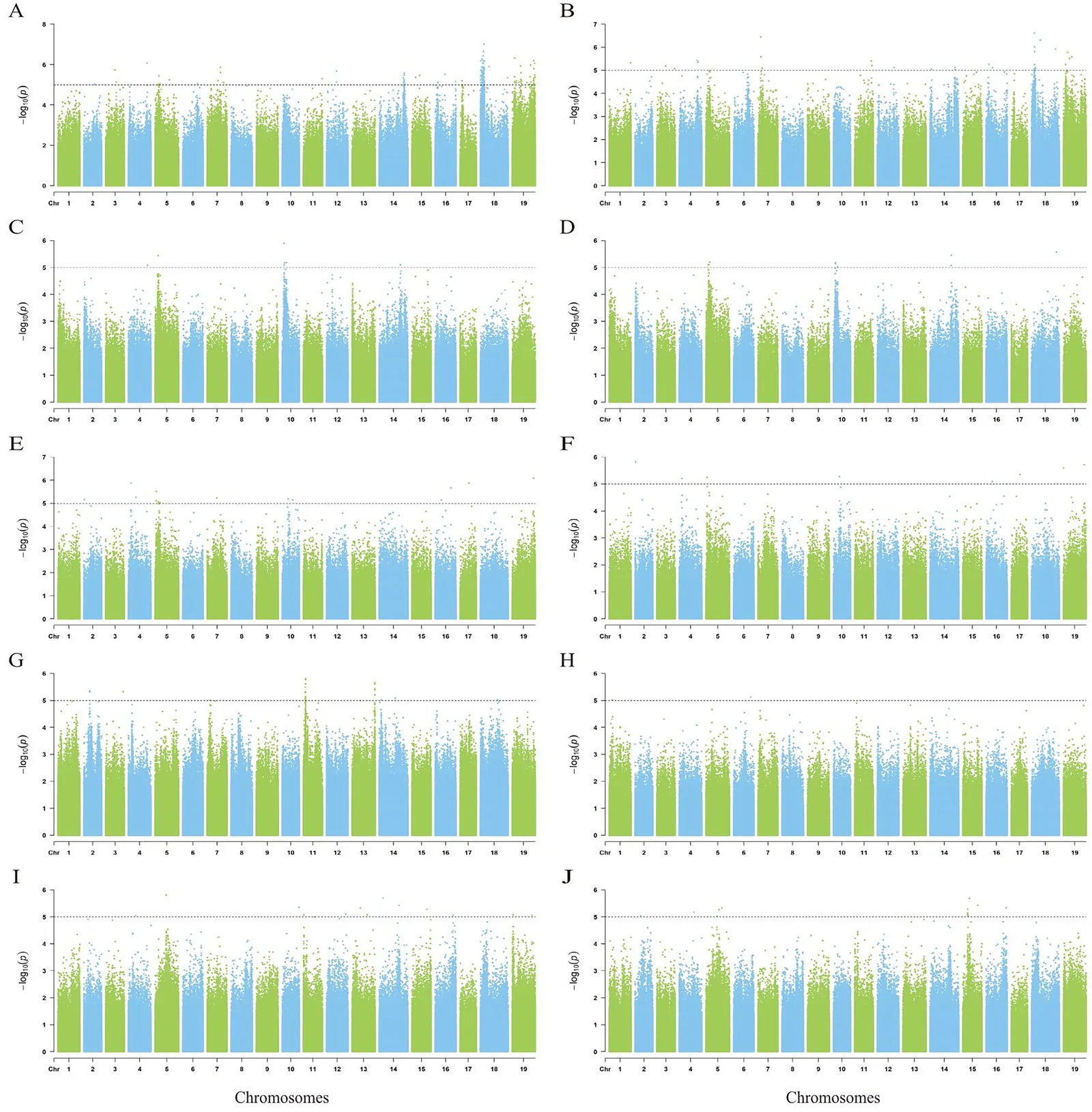
Manhattan plots of genome-wide association analysis for five grape leaf traits based on GLM. **(A, B)** Mature leaf size (YDX) in 2023 **(A)** and 2024 **(B)**; **(C, D)** serration length-to-width ratio (JCB) in 2023 **(C)** and 2024 **(D)**; **(E, F)** serration morphology (JCXZ) in 2023 **(E)** and 2024 **(F)**; **(G, H)** upper sinus overlapping type (SLPKD) in 2023 **(G)** and 2024 **(H)**; **(I, J)** leaf cross-sectional morphology (HJM) in 2023 **(I)** and 2024 **(J)**. The x-axis represents the physical position of SNPs on chromosomes, the y-axis shows −log_10_(P), and the horizontal dashed line indicates the genome-wide significance threshold (−log_10_(P) = 5).

To identify high-confidence SNPs stably associated with the traits, we screened for significant loci reproducibly detected across both years within each of the three models (**Table 2**). The models differed substantially in their ability to detect loci reproducible across both years. FarmCPU showed the highest detection power, identifying 18, 10, 27, 13, and 2 reproducible SNPs for YDX, JCB, JCXZ, SLPKD, and HJM, respectively. GLM showed moderate performance, detecting 15, 4, 7, and 1 reproducible SNPs for YDX, JCB, JCXZ, and HJM, respectively, with no reproducible significant loci detected for SLPKD. MLM had the lowest detection power, with only seven reproducible SNPs detected, all for JCXZ.

**Table 2:** Statistics of stable SNP loci co-detected in 2023 and 2024.

Model	Trait	Number of SNPs
MLM	YDX	0
	JCB	0
	JCXZ	7
	SLPKD	0
	HJM	0
GLM	YDX	15
	JCB	4
	JCXZ	7
	SLPKD	0
	HJM	1
FarmCPU	YDX	18
	JCB	10
	JCXZ	27
	SLPKD	13
	HJM	2

Among the five high-stability traits subjected to GWAS, JCXZ was the only trait for which significant SNPs were reproducibly detected by all three models in both years. Given its biological importance as a defining feature of grapevine leaf morphology, we selected JCXZ for subsequent candidate gene analysis. The multi-model and multi-year reproducibility thus provided statistical support for this selection, although reproducibility was not the sole criterion. These high-confidence loci, stably detected across years and models, provide a robust genetic basis for identifying candidate genes. Accordingly, subsequent analyses focused on JCXZ, with functional annotation and expression analysis of candidate genes within its significantly associated genomic regions.

### 3.3 Candidate genes for leaf serration morphology identified from stable loci

Building on the stable association signals identified above, we next sought to resolve candidate genes responsible for leaf serration variation through functional annotation and expression validation. We searched for candidate genes within ±15 kb genomic regions flanking the stable SNP loci (**Supplementary Table S6**). The number of stable loci and corresponding candidate genes differed among models: GLM detected 7 stable loci, from which 10 candidate genes were identified (0–4 per locus). MLM also detected 7 stable loci, yielding 10 candidate genes (the same 7 loci as GLM). FarmCPU detected 27 stable loci (some with no candidate genes), yielding 59 candidate genes (0–7 per locus).

Seven stable SNP loci were detected by all three models. Of these, candidate genes were identified within the ±15 kb regions flanking four loci (marker.321541, marker.783052, marker.2652352, and marker.5422047), whereas no candidate genes were found for the remaining three loci (marker.1088264, marker.4376037, and marker.4656403). These four loci corresponded to 10 candidate genes: *VIT_02s0025g00450*, *VIT_02s0025g00470*, *VIT_04s0008g03460*, *VIT_04s0008g03440*, *VIT_04s0008g03450*, *VIT_10s0003g03710*, *VIT_19s0027g01770*, *VIT_19s0027g01790*, *VIT_19s0027g01800*, and *VIT_19s0027g01780*. Seven of these genes had functional annotations (**Supplementary Table S7**). The remaining three (*VIT_04s0008g03450*, *VIT_19s0027g01800*, and *VIT_19s0027g01780*) were annotated as uncharacterized proteins and were therefore not included.

To assess the functional relevance of the ten candidate genes, we retrieved KEGG pathway annotations and combined them with functional annotations (**Supplementary Table S7**). The candidate genes mapped to pathways including plant hormone signal transduction, MAPK signaling, ubiquitin-mediated proteolysis, and RNA degradation (**Figure 4**). These pathways are broadly associated with plant development, but their specific roles in leaf serration morphogenesis remain to be validated experimentally. Based on pathway and functional annotations, we selected four candidate genes most relevant to leaf development for expression analysis: *VIT_04s0008g03460* (encoding a histidine kinase, involved in two-component signal transduction), *VIT_10s0003g03710* (encoding Cullin-4, involved in ubiquitin-mediated proteolysis), *VIT_02s0025g00470* (containing an SP-RING domain, with potential ubiquitin ligase activity), and *VIT_04s0008g03440* (encoding a homolog of the exosome complex nuclease RRP46, involved in RNA degradation and processing) (**Supplementary Table S7**). The remaining six candidate genes were excluded from expression analysis: three were uncharacterized proteins, and the other three had annotations less directly related to leaf development.

**Figure 4:**
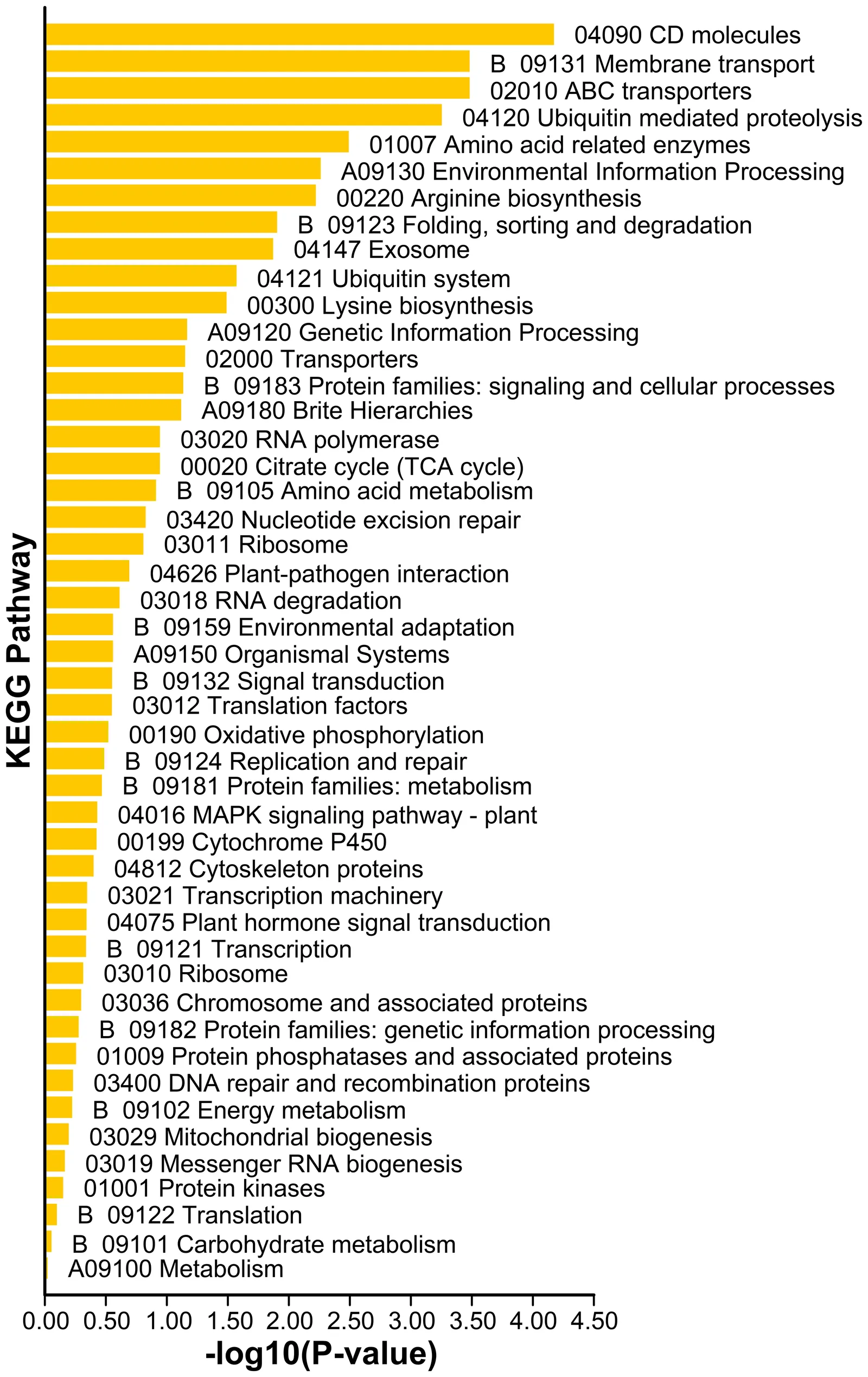
KEGG pathway enrichment analysis of the candidate genes. The x-axis indicates -log10(P-value) for each enriched pathway, and the y-axis lists the KEGG pathway names. Pathways are ranked by enrichment significance.

### 3.4 Differential expression of candidate genes across serration types

Nine grapevine accessions representing three serration phenotypes (mixed-type, straight-sided, and convex-sided; three accessions per phenotype) were used for qRT-PCR validation (**Figure 5**). qRT-PCR analysis revealed that the four candidate genes showed significant differential expression among these accessions (**Figure 6**). *VIT_10s0003g03710* and *VIT_02s0025g00470* showed highly similar expression patterns, with the highest expression in straight-sided serrations (means of 1.87 and 1.83, respectively), significantly higher than in mixed-type (means of 1.06 and 1.00, respectively) and convex-sided serrations (means of 0.61 and 0.31, respectively). In contrast, *VIT_04s0008g03460* showed the highest expression in convex-sided serrations (mean = 1.72) and the lowest in straight-sided serrations (mean = 0.70). *VIT_04s0008g03440* did not follow a clear pattern: its expression showed no significant correlation with any specific serration type, ranging from 0.77 to 1.31 across groups.

**Figure 5:**
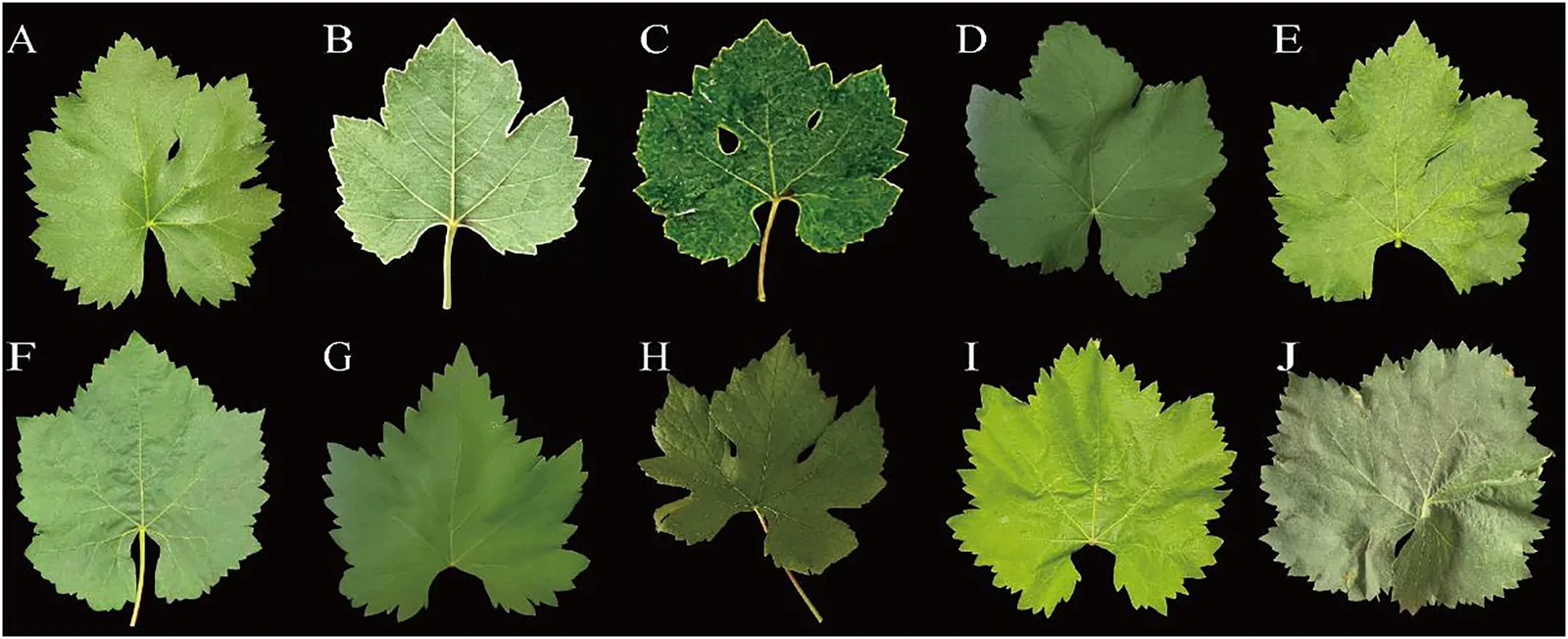
Representative leaf images of the nine grapevine accessions used for qRT-PCR validation and the reference accession ‘Yinhong’. Accessions were assigned to three serration-type groups (mixed, straight-sided, and convex-sided) according to the national agricultural industry standard NY/T 2563-2014. **(A–C)** Accessions classified as mixed-type; **(D–F)** accessions classified as straight-sided; **(G–I)** accessions classified as convex-sided; **(J)** reference accession ‘Yinhong’ (convex-sided). Note that within-leaf and among-leaf variation in individual serration morphology may exist; the images are shown for accession identification rather than as a definition of the classification categories.

**Figure 6:**
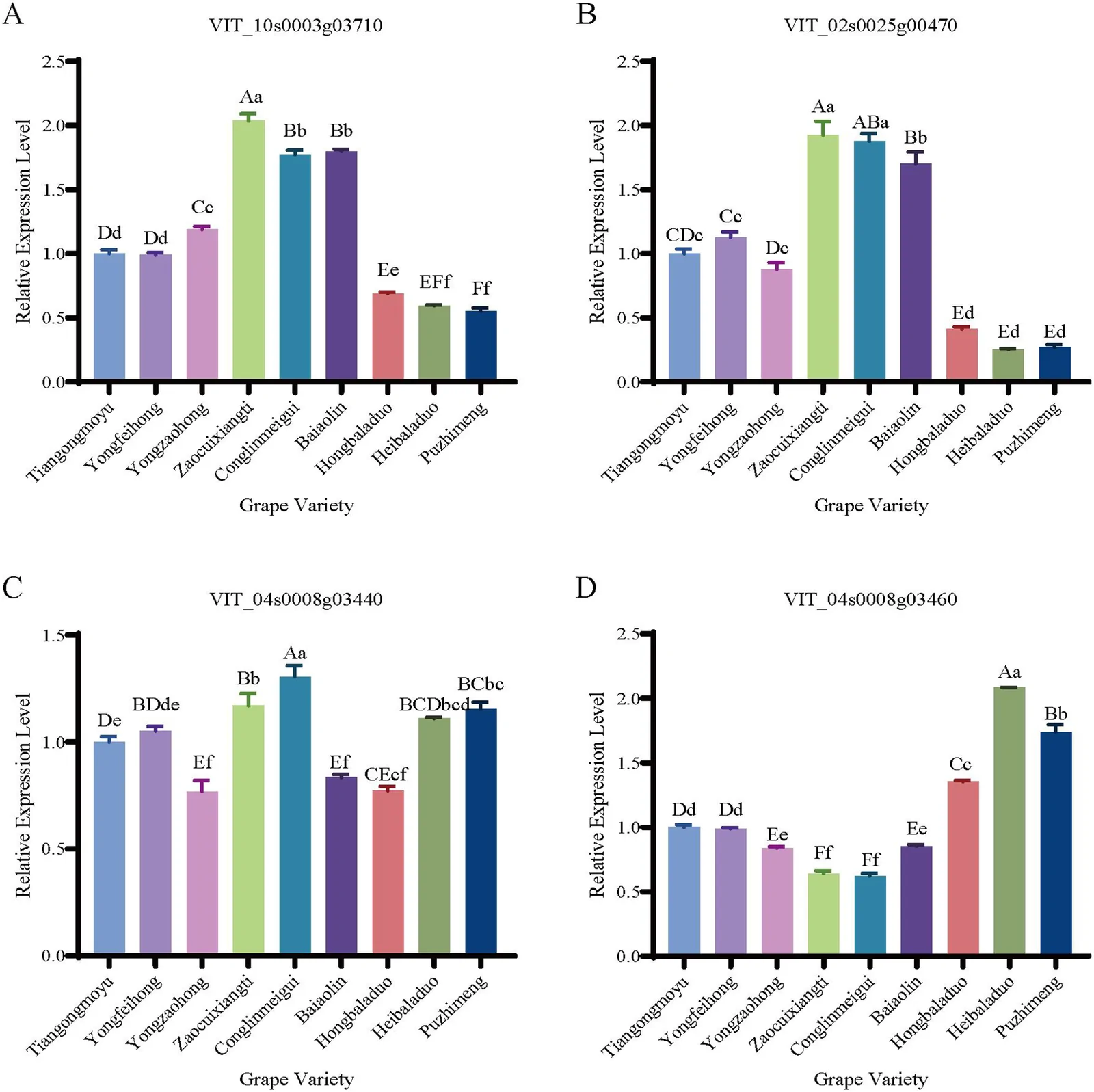
Relative expression levels of four candidate genes in nine grapevine accessions with contrasting leaf serration phenotypes. **(A)** *VIT_10s0003g03710*; **(B)** *VIT_02s0025g00470*; **(C)** *VIT_04s0008g03440*; **(D)** *VIT_04s0008g03460*. The x-axis displays nine individual grapevine accessions colored by cultivar. Their grouping by serration phenotype (mixed-type: ‘Tiangongmoyu’, ‘Yongfeihong’, ‘Yongzaohong’; straight-sided: ‘Zaocuixiangti’, ‘Conglinmeigui’, ‘Baiaolin’; convex-sided: ‘Hongbaladuo’, ‘Heibaladuo’, ‘Puzhimeng’; three accessions per group) is indicated by horizontal brackets above the x-axis labels. The y-axis represents relative expression levels normalized to the *Vvi-ACT* reference gene. Error bars indicate the range of three technical replicates. Different uppercase and lowercase letters above bars denote significant differences at P < 0.01 and P < 0.05, respectively, as determined by one-way ANOVA followed by Tukey’s HSD test. Leaf samples used for qRT-PCR were fully expanded mature leaves collected from the 7th–9th nodes, consistent with the sampling scheme used for phenotypic characterization.

Tissue-specific expression analysis revealed distinct expression patterns of the four candidate genes across grapevine tissues, although all were highly expressed in young leaves (**Figure 7**). *VIT_04s0008g03440* and *VIT_04s0008g03460* showed similar patterns, with the highest expression in young leaves (3.48 and 2.54, respectively), followed by stems (1.96 and 1.38, respectively), and the lowest in fruits (0.13 and 0.05, respectively). *VIT_10s0003g03710* was highly expressed in both young leaves (1.85) and tendrils (1.66). *VIT_02s0025g00470* showed stronger tissue specificity: its expression in stems (4.36) was nearly equal to that in young leaves (4.29), and both were significantly higher than in other tissues, indicating high expression in both stem and leaf tissues.

**Figure 7:**
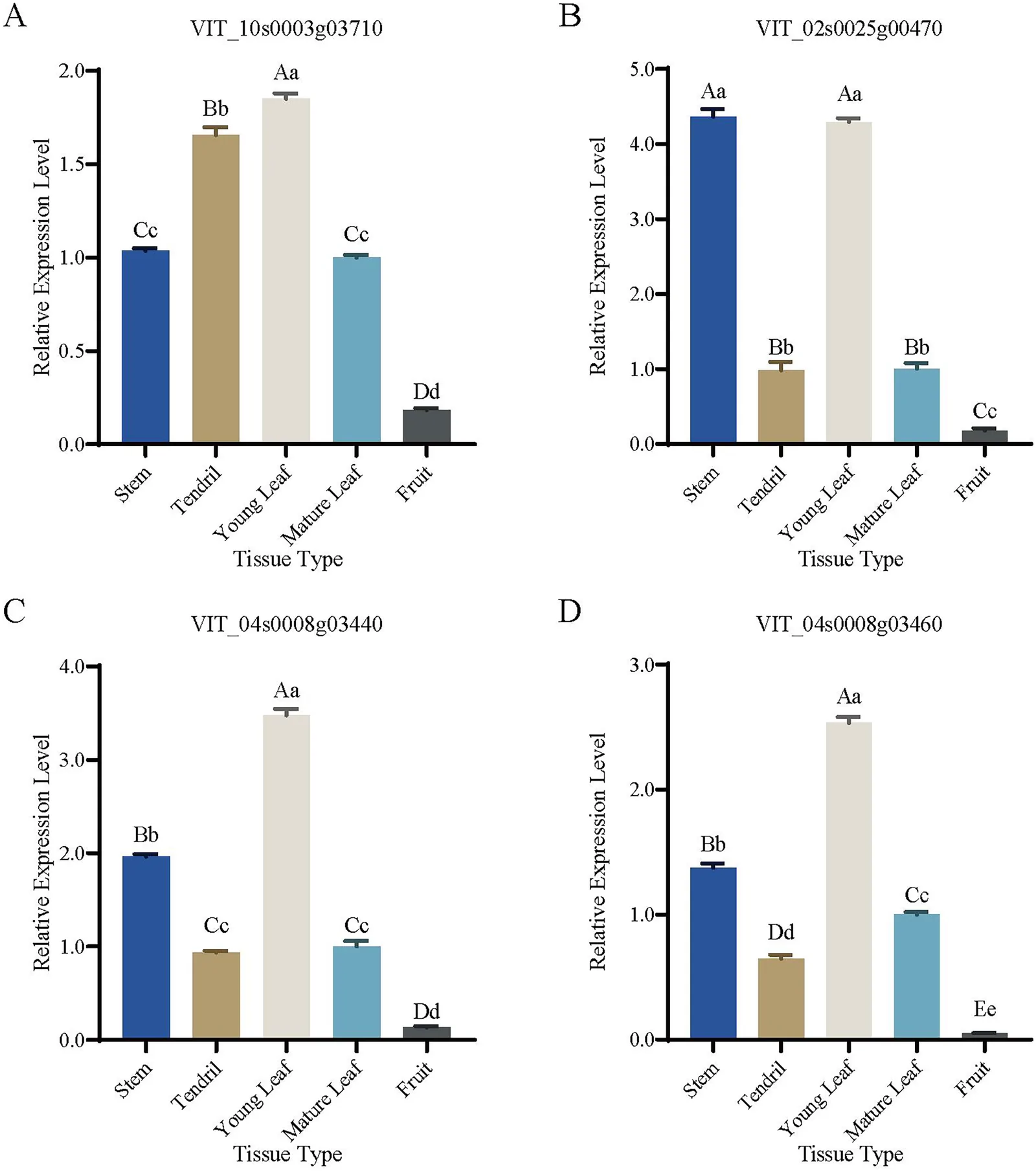
Tissue-specific expression patterns of the four candidate genes in ‘Yinhong’ grapevine. **(A)** *VIT_10s0003g03710*; **(B)** *VIT_02s0025g00470*; **(C)** *VIT_04s0008g03440*; **(D)** *VIT_04s0008g03460*. The x-axis displays five tissue types: tendrils, stems, young leaves, mature leaves, and fruits. The y-axis represents relative expression levels normalized to the *Vvi-ACT* reference gene. Error bars indicate the range of three technical replicates. Different lowercase letters above bars denote significant differences among tissues at P < 0.05, as determined by one-way ANOVA followed by Tukey’s HSD test.

In summary, all four candidate genes were highly expressed in young leaves. *VIT_10s0003g03710* and *VIT_02s0025g00470* were most highly expressed in straight-sided serrations, *VIT_04s0008g03460* was most highly expressed in convex-sided serrations, and *VIT_04s0008g03440* showed no significant correlation with serration phenotype.

## 4 Discussion

Leaf morphology is a core agronomic trait in grapevine, influencing light interception, water use efficiency, and adaptation to abiotic stress (Zhu et al., 2010). Through source–sink interactions, it also affects fruit yield and quality, making it a key target for genetic improvement (Zhang et al., 2024). Leaf morphogenesis involves complex developmental regulation and phenotypic plasticity (Tsukaya, 2014). In this study, we initially evaluated ten mature leaf morphological traits in 153 grapevine accessions and selected five traits with high interannual stability for GWAS. We identified genetic loci and candidate genes associated with leaf serration morphology (JCXZ) and used expression analysis to explore their possible roles.

Interannual stability is a prerequisite for accurate genetic mapping. Five traits—YDX, JCB, JCXZ, SLPKD, and HJM—showed interannual correlation coefficients ≥ 0.8, indicating strong genetic control and suitability for GWAS (Barbagallo et al., 2020; Flutre et al., 2022). These results are consistent with previous findings in grapevine (Barbagallo et al., 2020; Flutre et al., 2022; Zhang et al., 2024). The remaining five traits showed lower stability, likely due to environmental and small-effect polygenic regulation (Flutre et al., 2022).

To mitigate population structure bias and spurious associations, we adopted three complementary statistical models—GLM, MLM, and FarmCPU—to improve the reliability of association signals (Liu et al., 2016; Tamba et al., 2017). Across the five high-stability traits, 468 significant SNP loci were detected over two years. Among these traits, JCXZ was the only one with reproducible signals detected by all three models in both years, providing a robust basis for candidate gene screening. FarmCPU detected more stable signals than GLM and MLM, likely because it better controls population structure and kinship (Liu et al., 2016; Ali et al., 2025). The differences among models highlight the value of multi-model cross-validation, consistent with previous reports (Ali et al., 2025; Gan et al., 2026).

Compared with a previous GWAS of 25 grapevine leaf traits that used a single model and single-year phenotypic data (Zhang et al., 2024), our study combined multi-model cross-validation with two-year phenotypic data and focused specifically on JCXZ. This strategy improved signal reliability and identified previously unreported candidate genes (e.g., *VIT_04s0008g03460* and *VIT_10s0003g03710*). Similar advantages of multi-model approaches have been reported in mango GWAS (Ali et al., 2025).

The ten leaf traits examined here are likely governed by partially distinct developmental processes. Leaf size is mainly determined by cell proliferation and expansion, whereas serration traits depend on localized cell division and differentiation at the leaf margin. Our phenotypic correlation analysis showed that leaf size and serration traits were not consistently correlated, suggesting distinct genetic networks. Therefore, the loci identified for JCXZ may represent regulators of marginal tissue development rather than general leaf growth.

KEGG annotation of the ten JCXZ-associated candidate genes revealed enrichment in plant hormone signal transduction, ubiquitin-mediated proteolysis, and RNA degradation pathways (**Supplementary Table S7**). These pathways are broadly associated with plant development (Conklin et al., 2018), but their specific role in leaf serration remains to be determined. By integrating transcriptome data with qRT-PCR, we identified four genes preferentially expressed in young leaves and correlated with serration phenotypic differences. *VIT_04s0008g03460* encodes a histidine kinase involved in cytokinin signal transduction (Schaller et al., 2011). *VIT_10s0003g03710* encodes Cullin-4, a core subunit of Cullin-RING ubiquitin ligase complexes that regulate organ development (Sharma et al., 2016; Zhang et al., 2017). *VIT_02s0025g00470* contains an SP-RING domain with potential E3 ubiquitin ligase activity (Stone et al., 2005). *VIT_04s0008g03440* encodes the exosome component RRP46, involved in RNA degradation and quality control (Januszyk and Lima, 2014). These genes belong to multi-member families in grapevine, suggesting possible functional redundancy. Several associated SNPs were located in putative promoter or intronic regions, implying possible regulatory effects on transcript abundance rather than protein sequence. However, whether these variants affect gene expression or protein function remains to be determined. Members of these gene families have been implicated in leaf development in other plant species (Bernhardt et al., 2006; Wu et al., 2021); however, their specific roles in grapevine leaf serration require direct functional validation.

The expression patterns observed here are consistent with, but do not prove, the involvement of these genes in serration morphogenesis. Cullin-4 and SP-RING-type protein were more highly expressed in straight-sided serrations, whereas histidine kinase was more highly expressed in convex-sided serrations. These differences suggest that ubiquitin–proteasome activity and cytokinin signaling may contribute to serration variation, although the associations are correlative. The candidate genes are involved in hormone signaling, protein degradation, and RNA metabolism—core processes in plant development—and may form a regulatory network relevant to leaf morphogenesis. This interpretation is consistent with previous reports (Conklin et al., 2018; Zhang et al., 2024), but direct functional validation in grapevine is required.

Several limitations should be acknowledged. First, the sample size of 153 accessions may limit detection of low-frequency alleles (Flutre et al., 2022; Ali et al., 2025). We mitigated this by using multi-model cross-validation and two-year replication to focus on stable, high-confidence loci. Second, categorical scoring of complex traits may reduce phenotypic resolution. Third, functional annotation relies largely on homology-based inference. Fourth, functional validation was limited to transcript-level analysis, without protein-level or gain-/loss-of-function experiments. Fifth, phenotypic data were collected from a single location over two years, limiting genotype-by-environment analysis. Furthermore, conventional canopy management is likely to remain the primary approach for leaf morphology improvement in the near term.

Despite these limitations, this study provides new insights into the genetic dissection of leaf serration morphology in grapevine. Future research should expand the population size, improve phenotyping resolution, and validate candidate gene functions through transgenic or genome-editing approaches. Leaf serration depth and density have been shown to differentiate along climatic gradients and may contribute to stress tolerance in some plant species (Royer et al., 2008); whether this applies to grapevine requires further investigation. The JCXZ-associated candidate genes identified here are mostly involved in signal transduction and stress responses, suggesting a potential link between leaf serration morphology and stress tolerance. Future studies should integrate physiological measurements to verify differences in photosynthetic efficiency and stress tolerance among accessions with different serration morphologies. Such knowledge may inform future genetic improvement strategies, although practical application will require careful evaluation of costs and complexity.

## 5 Conclusions

We integrated two consecutive years of phenotypic data, multi-model genome-wide association analysis, and gene expression validation to characterize seven high-confidence SNP loci associated with leaf serration morphology in a panel of 153 grapevine accessions and identify three candidate genes showing differential expression associated with serration morphological differentiation. Our expression data suggest that the ubiquitin–proteasome pathway (represented by Cullin-4 and SP-RING-type protein) and cytokinin two-component signaling (represented by histidine kinase) may be associated with leaf serration variation; however, the causal relationships and underlying mechanisms remain to be functionally validated. These findings advance our understanding of the genetic architecture of leaf serration morphological variation in grapevine, provide candidate loci for future functional studies, and may inform genetic improvement strategies for leaf morphology, although any practical application would require further experimental support.

## Data Availability

The datasets presented in this study can be found in online repositories. The names of the repository/repositories and accession number(s) can be found in the article/**Supplementary Material**.
